# The Effect of Vitamin D Adequacy on Thyroid Hormones and Inflammatory Markers after Bariatric Surgery

**DOI:** 10.3390/metabo13050603

**Published:** 2023-04-27

**Authors:** Roberta França, Adryana Cordeiro, Silvia Elaine Pereira, Carlos José Saboya, Andrea Ramalho

**Affiliations:** 1 Faculty of Medicine, Federal University of Rio de Janeiro (UFRJ), Rio de Janeiro 21941-598, Brazil; 2 Micronutrients Research Center (NPqM), Institute of Nutrition, Federal University of Rio de Janeiro (UFRJ), Rio de Janeiro 21941-902, Brazil; 3 Multidisciplinary Center of Bariatric and Metabolic Surgery Carlos Saboya, Rio de Janeiro 22280-020, Brazil; 4 Department of Social Applied Nutrition, Institute of Nutrition Josué de Castro, Federal University of Rio de Janeiro (UFRJ), Rio de Janeiro 21941-902, Brazil

**Keywords:** vitamin D, thyroid hormones, inflammation, bariatric surgery, weight loss

## Abstract

Vitamin D status affects the clinical and corporal outcomes of postoperative patients who undergo a Roux-en-Y gastric bypass (RYGB). The aim of this study was to evaluate the effect of adequate vitamin D serum concentrations on thyroid hormones, body weight, blood cell count, and inflammation after an RYGB. A prospective observational study was conducted with eighty-eight patients from whom we collected blood samples before and 6 months after surgery to evaluate their levels of 25-hydroxyvitamin D 25(OH)D, thyroid hormones, and their blood cell count. Their body weight, body mass index (BMI), total weight loss, and excess weight loss were also evaluated 6 and 12 months after surgery. After 6 months, 58% of the patients achieved an adequate vitamin D nutritional status. Patients in the adequate group showed a decrease in the concentration of thyroid-stimulating hormone (TSH) (3.01 vs. 2.22 µUI/mL, *p* = 0.017) with lower concentrations than the inadequate group at 6 months (2.22 vs. 2.84 µUI/mL, *p* = 0.020). Six months after surgery, the group with vitamin D adequacy showed a significantly lower BMI compared with the inadequate group at 12 months (31.51 vs. 35.04 kg/m^2^, *p* = 0.018). An adequate vitamin D nutritional status seems to favor a significant improvement in one’s thyroid hormone levels, immune inflammatory profile, and weight loss performance after an RYGB.

## 1. Introduction

The prevalence of obesity has increased worldwide in the past 50 years. It represents a major nutritional problem [1]. Drug resources cannot completely resolve the disorders associated with higher body mass index (BMI) values [2]. In this case, bariatric surgery can be an effective intervention for the prevention and remission of associated disorders [3]. However, vitamin and mineral deficiencies are common conditions after surgical procedures that use disabsorptive, restrictive, and mixed techniques [4]. 

In this context, vitamin D deficiencies represent a considerable problem in the preoperative and postoperative period in patients with obesity who undergo bariatric surgery [5,6,7]. Considering the adequacy of vitamin D > 30 ng/mL, 25-hydroxyvitamin D (25(OH)D) serum concentrations can be evaluated to optimize bone health, prevent secondary hyperparathyroidism, and improve extraskeletal functions such as immunomodulatory and metabolic ones [4].

Vitamin D plays a role in the immune system, is positively associated with higher T lymphocytes concentrations, and is related to the prevention of inflammatory processes [8]. The excess of body weight in pre-surgical period and the surgical procedure itself that provides intense weight loss, it is important in the investigation of the role of vitamin D in the immune and inflammatory system under these conditions [8].

In addition, the inflammatory response and the patient’s nutritional status can favor alterations on the pituitary–thyroid axis that can lead to diseases [9]. Subclinical hypothyroidism, defined as an increase in serum thyroid-stimulating hormone (TSH) with normal serum-free thyroxin (FT4) concentrations, affects approximately 10% of the adult population. This condition can progress to obvious hypothyroidism and is likely caused by autoimmune thyroiditis (Hashimoto) [10], but it can also have other causes.

Clinical evidence supports an association between one’s vitamin D nutritional status and autoimmune thyroid disorders such as Hashimoto’s thyroiditis [11], but the effect of this nutrient on thyroid function during intense weight loss, which can potentialize the prevention of disorders such as subclinical hypothyroidism as an adjunctive treatment, has been little explored.

Thus, this study aimed to evaluate the effect of adequate vitamin D serum concentrations (≥30 ng/mL) on the immune and inflammatory profile and thyroid hormones after intense weight loss due to bariatric surgery.

## 2. Methods

A prospective observational study was performed on 90 patients with obesity who underwent an elective Roux-en-Y gastric bypass (RYGB) surgery. These participants were recruited from a medical clinic specializing in obesity control in Rio de Janeiro, Brazil, from June 2019 to April 2022. Of the 90 enrolled patients, 2 were withdrawn from the study due to lost follow-up data. No adverse effects were reported by any of the participants. This study was approved by the Research Ethics Committee of the Hospital Universitário Clementino Fraga Filho, Federal University of Rio de Janeiro, Brazil (Research Protocol number 011/10—CEP) and complied with the recommendations of the Declaration of Helsinki. Informed consent was obtained from all patients included in this study.

The inclusion criteria were as follows: patients with BMI ≥ 35 kg/m^2^, aged ≥ 20 and <60 years, and serum biochemistry outcomes monitored before their scheduled RYGB. The exclusion criteria were as follows: acute and chronic infections with criteria characterizing severity, evidence of any disease affecting the immune system balance, multiple sclerosis, inflammatory bowel disease and other autoimmune diseases, abnormalities of liver enzyme function, previous bariatric procedure, immunosuppressive therapy, pregnancy or in lactation period or planning a pregnancy in the next 6 months, smoking habits, cancer diagnosis, use of anticonvulsant medications or drugs known to interfere with vitamin D metabolism, current insulin treatment, and consumption of vitamin D supplements within 6 months prior to blood work.

The patients underwent a routine anthropometric evaluation (body weight and height), medical history assessment, and biochemical analyses of blood samples before the surgery and 6 months after the bariatric surgery. Their body weight, BMI calculation, total weight loss, and excess weight loss were also evaluated at 6 and 12 months after the surgery.

In the postoperative period, a standard supplementation protocol of daily vitamins and minerals was implemented at the institution and was prescribed by the medical doctors for all the patients. The vitamin D supplementation was administered as vitamin D3 (cholecalciferol) isoform in a dosage of 3500 IU/week. The patients had consultations with medical doctors and dietitians during the pre- and postoperative RYGB period. In all the patient consultations, the importance of the daily use of the vitamins and mineral supplements and lifestyle advice were emphasized, and educational material was provided.

Each participant’s weight (kg) and height (m) were measured to calculate their BMI (kg/m^2^). After surgery, the percentage of excess weight loss (%EWL) and the percentage of total weight loss (%TWL) were evaluated [12,13]. %EWL = (100 × (initial weight − actual weight)) (initial weight − ideal weight), where the ideal body weight is equivalent to a BMI of 25 kg/m^2^. %TWL = ((initial weight − actual weight) ÷ actual weight) × 100.

All the subjects provided an overnight 12 h fasting blood sample, and laboratory measurements were performed in certified laboratories associated with the Center for Bariatric and Metabolic Surgery (Rio de Janeiro, Brazil).

An analysis of the serum concentrations of 25(OH)D was conducted by using the High-Performance Liquid Chromatography method with an ultraviolet detector. The serum TSH, total triiodothyronine (T3), and free thyroxine (FT4) (normal range: 0.7–1.48 ng/dL) were measured via a chemiluminescence immunoassay to evaluate the thyroid hormones. A diagnosis of hypothyroidism was defined as the presence of persistently elevated levels of thyroid-stimulating hormone (TSH) (4.6–19.9 mIU/L) with free thyroxine (T4) within the reference range.

Laboratory tests were performed to assess the cell blood count (leukocytes, neutrophils, lymphocytes, and platelets) as an immunological parameter and were determined using an automated assay and optical microscopy. The neutrophil-to-lymphocyte ratio (NLR) was determined as an inflammatory marker of low-grade inflammation. In the same way, the leukocyte count was also considered an inflammatory parameter.

Furthermore, serum uric acid and high-sensitivity C-reactive protein (hs-CRP) were evaluated as biochemical variables associated with intensive weight loss and were measured by using the colorimetric enzyme method and immunoturbidimetry, respectively.

Serum concentrations of parathyroid hormone (PTH) and calcium were also evaluated to assess the clinical biomarkers of vitamin D bone metabolism and were determined via a chemiluminescence immunoassay and colorimetric assay, respectively.

Six months after surgery, the patients were divided into two groups: the adequate vitamin D status group (AS group) and the inadequate vitamin D status group (IS group). To classify the vitamin D adequacy of the patients in our study, we used the Endocrine Society criteria, whereby 25(OH)D serum levels ≥ 30 ng/mL are considered “sufficient” [14], levels between 20.1 and 29.9 ng/mL are considered “insufficient”, and levels ≤ 20 ng/mL are “deficient”. According to such criteria, in this study, “sufficient” was considered adequate, and “insufficient” and “deficient” were considered inadequate.

Data related to the habit of practicing physical exercise, such as the type, time (in years and minutes/week), and weekly frequency (days/week) were collected through a questionnaire previously prepared during the first consultation [15].

The required sample size was determined according to the main aim of the study, which was to investigate the effect of adequate vitamin D serum concentrations (≥30 ng/mL) on the immune and inflammatory profile and thyroid hormones after intense weight loss due to bariatric surgery. The following parameters were assumed: use of bilateral tests, a level of significance of 5%, a statistical power of 80%, and an expected correlation of −0.25.

According to the sample calculation, 80 individuals were required. The sample size value was inflated by 10% to anticipate possible losses.

The primary outcome measure was the between group difference in serum concentrations of 25(OH)D (ng/mL) on the immune and inflammatory profile and thyroid hormones after intense weight loss due to bariatric surgery.

All data are expressed as the mean (standard deviation (SD)) for continuous variables and as percentages of numbers for categorical variables. The normality of the variables was tested by using the Kolmogorov–Smirnov test.

To characterize the association between 25(OH)D serum status and thyroid, immune, and inflammatory biochemical parameters, comparisons were made between and within groups at baseline and follow up by using an intent-to-treat analysis.

A Student’s two-sample *t*-test was used to evaluate the differences between the groups at baseline and after severe weight loss for the data with a normal distribution, and a Mann–Whitney U test was used for variables with a non-normal distribution. To assess the same group of patients before and after surgery, a Student’s paired *t*-test was used for the parametric variables and a Wilcoxon signed-rank test was used for the nonparametric variables. Qualitative data were evaluated by using a Chi-square test. A *p* value < 0.05 was considered statistically significant. Statistical analyses were performed by using the Statistical Package for the Social Sciences software (SPSS, version 21).

## 3. Results

In total, 88 patients completed the study: 51 (58%) were in the AS group (≥30 ng/mL) and 37 (42%) were in the IS group (<30 ng/mL).

At baseline, the 25(OH)D serum concentration, mean age, sex, and BMI were not significantly different between the two groups (Table 1). Likewise, at baseline, the investigated variables (Table 2 and Table 3) did not differ significantly between the two groups.

At baseline, 16 patients (18.2%) had sufficient 25(OH)D (≥30 ng/mL) levels without changes occurring between the groups, and 13 patients (14.8%) had hypothyroidism without changes occurring between the groups (Table 1).

**Figure 1:**
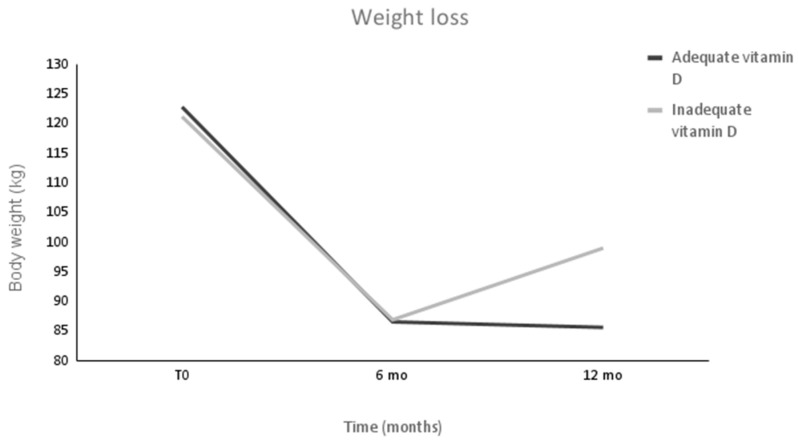
Patients’ body weight after intense weight loss for 12 months. Values are expressed as means. *n* = 88. Values were measured in both groups before surgery (T0) and at 6 and 12 months after surgery (6 and 12 mo). Pre- and postsurgery values were significantly different in both groups at all time intervals compared to the baseline with differences between groups at 12 mo.

Patients maintained their usual caloric expenditure without changing their physical activities up to 6 months after surgery. At this time (6 months), both groups showed a significant reduction in BMI (Δ = −13.03 (2.79) kg/m^2^, *p* < 0.001 in the AS group; Δ = −12.61 (3.6) kg/m^2^, *p* < 0.001 in the IS group) with no significant differences between them. At 12 months after surgery, both groups maintained a significant decrease in body weight (Δ = −11.77 (4.64) kg/m^2^, *p* < 0.001 in the AS group; Δ = −8.81 (5.99) kg/m^2^, m^2^, *p* < 0.001 in the IS group); however, the AS group had a significantly lower BMI than the IS group (*p* = 0.018) (Table 4) (Figure 1).

Significant differences were observed in 25(OH)D levels between the groups in the period mentioned above (*p*< 0.001). The 25(OH)D serum concentrations increased significantly in the AS group (Δ = +16.08 (13.46) ng/mL, *p* < 0.001) without changes occurring in the IS group (Δ = +1.75 (8.33) ng/mL, *p* = 0.23). According to these results, the PTH serum levels decreased significantly only in the AS group (Δ = −5.27 (16.11) pg/mL, *p* = 0.04) without changes occurring between the groups, while the serum calcium levels were significantly higher in the AS group compared with the IS group 6 months after surgery (*p* = 0.008) (Table 2). Both groups showed a significant reduction in BMI (Δ = −13.03 (2.79) kg/m^2^, *p* < 0.001 in the AS group; Δ = −12.61 (3.61) kg/m^2^, *p* < 0.001 in the IS group) and a percentage of excess weight loss (% EWL) greater than 50% (Δ = −69.94 (15.62) %, *p* < 0.001 in the AS group; Δ = −69.75 (20.27) %, *p* < 0.001 in the IS group). There were no changes between the groups at 6 months postsurgery.

**Figure 2:**
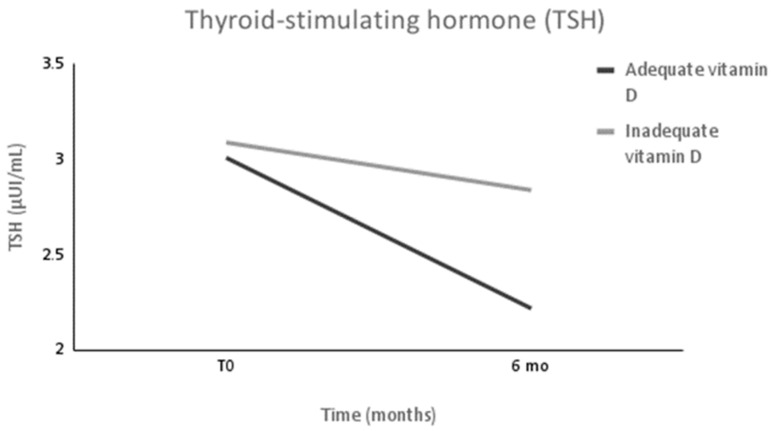
The thyroid-stimulating hormone (TSH) levels in subjects after expressive weight loss over 6 months. Values are expressed as means. *n* = 88. Values were measured for both groups before surgery (T0) and 6 months after surgery (6 mo). Intervals are significantly different only in the adequate vitamin D status group (AS group) with changes occurring between groups at 6 mo.

Thyroid hormone profiles are displayed in Table 2. Six months after surgery, the TSH levels were significantly lower in the AS group compared with the IS group (*p* = 0.026), in addition to decreasing significantly only in the AS group after surgery (Δ = −0.79 (2.09) µUI/Ml, *p* = 0.017) (Figure 2). The T3 levels decreased significantly in both groups without differences occurring between them. No statistical differences in FT4 were observed within each group or between the groups.

Data on the immunity and inflammatory parameters are presented in Table 3. Both groups showed a significant reduction in uric acid (Δ = −0.79 (1.49) mg/dL, *p* < 0.001 in the AS group; Δ = −0.78 (1.27) mg/dL, *p* = 0.001 in the IS group) and neutrophil count (Δ = −1040.74 (1413.21) mm^3^, *p* < 0.001 in the AS group; Δ = −829.09 (906.80) mm^3^, *p* = 0.009 in the IS group) without changes between groups.

**Figure 3:**
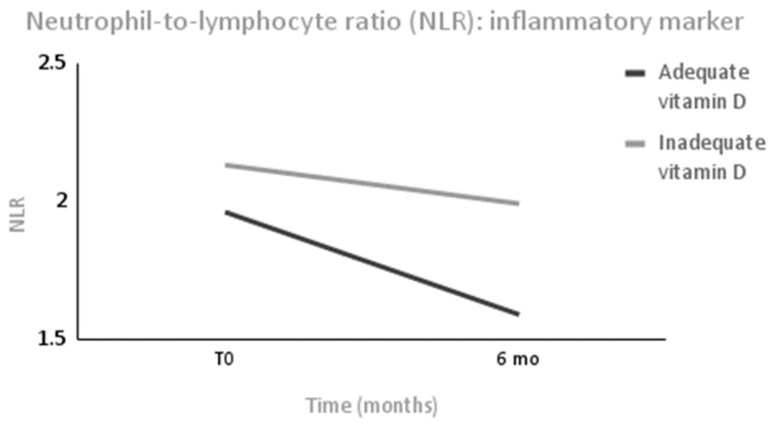
The neutrophil-to-lymphocyte ratio (NLR) in subjects after intense weight loss over 6 months. Values are expressed as means. *n* = 88. Values were measured for both groups before surgery (T0) and 6 months after surgery (6 mo). Intervals are significantly different only in the adequate vitamin D status group (AS group) without changes occurring between groups at T0 and 6 mo.

Patients in the AS group presented a higher lymphocyte count at 6 months compared with the IS group (*p* = 0.04). As inflammatory markers, leucocytes decreased only in the AS group (Δ = −1061 (1737) mm^3^, *p* < 0.001) without changes occurring between the groups, and NLR was significantly reduced only in the AS group (Δ = −0.37 (0.73), *p* = 0.01) without changes occurring between the groups after 6 months (Figure 3). The platelet cell count did not change within each group or between groups.

## 4. Discussion

The present study observed that as an adjuvant therapy, one’s vitamin D nutritional status can improve their thyroid hormone profile and immunoinflammatory parameters in patients with obesity who presented significant weight loss due to a surgical procedure. Vitamin D modulates the immune system by exerting vital effects on most of its cells, mainly because the vitamin D receptor (VDR) is expressed in lymphocytes, dendritic cells, and macrophages, and because of the presence of metabolizing hormones in immune cells. The active form of vitamin D (calcitriol) regulates inflammatory cytokine production and inhibits the proliferation of proinflammatory cells [16]. This seems to be a relevant contribution because, to our knowledge, this is the first study to evaluate this association in these conditions.

Our study demonstrated that TSH levels were lower in patients that had adequate vitamin D levels after bariatric surgery. A possible mechanism that could explain this association is the possibility that vitamin D may regulate TSH levels via receptors on pituitary thyrotropes and thyrocytes. Then, thyrocytes become less responsive to TSH stimulation in vitamin D deficiency status, leading to increased TSH levels which in turn may predispose to thyroid pathology [17].

The less inflammatory profile observed in patients that achieved an adequate vitamin D status in our study corroborated with this hypothesis. Some studies demonstrated that higher vitamin D levels are associated with lower TSH levels in euthyroid patients and that a relationship even exists between low vitamin D and higher thyroid volumes with higher TSH levels [17,18,19].

In this study, there was no association between vitamin D nutritional status and the FT4 hormone. Botelho et al. found a positive correlation between FT4 and vitamin D status only in patients with Hashimoto’s thyroiditis disease [20] and not in the control euthyroid group. Studies have suggested that the VDR polymorphisms may be associated with autoantibody production and disease susceptibility [21].

Few studies have attempted to investigate the correlation between 25(OH)D serum concentrations and hypothyroidism. One of them measured this metabolite in 30 hypothyroid patients and 30 healthy participants, and the authors discovered that hypothyroid patients had a vitamin D deficiency, which was significantly associated with the severity of the hypothyroidism [22]. Mirhosseini et al. showed in a large database that 25(OH)D serum concentrations ≥ 50 ng/dL were associated with a 30% reduced risk of overt hypothyroidism [23]. In this way, our study originally showed that achieving the recommended 25(OH)D serum concentrations above 30 ng/dL in association with an intense body weight loss could improve the clinical parameters of thyroid hormone function, which points to an adjuvant goal for sustainable weight loss.

In the present study, we observed that even when having the same weight loss performance, TSH levels were significantly lower only in the AS group, which led to a lower stimulation and receptor saturation on this hormonal axis, which prevents diseases such as subclinical hypothyroidism. Thus, it seems that the beneficial role of vitamin D in thyroid hormone functions is not restricted to its known role in Hashimoto’s autoimmune hypothyroidism.

Another finding analyzed in this study was that in patients who experienced significant weight loss 6 months after bariatric surgery, an adequate vitamin D nutritional status improved the NLR, which is an immune inflammatory marker, and the leukocyte count, which is an additional marker of systemic and low-grade inflammation [24]. The NLR was tested as a guide for the prognosis of various diseases, including sepsis, cancer, and coronavirus disease [25,26]. Supporting our findings, some studies showed the inverse association between vitamin D status and NLR [24,27,28]. A cohort study with adolescent girls found that a vitamin D supplementation of 50,000 IU/week for 3 months reduced the NLR distribution [27]. In this study, the dosage was lower, but there was a remission of the excess weight which was a proinflammatory factor, and this indicated that it was an appropriate dose for this clinical condition.

The less systemic inflammation observed in patients with an adequate vitamin D nutritional status in this study could support the conclusions of some meta-analyses of clinical trials and systematic reviews that have shown that vitamin D can prevent and improve the prognosis of viral respiratory tract infections [29,30,31]. By reducing the concentrations of proinflammatory cytokines via the innate immune system, vitamin D reduces the inflammation that injures the lining of the lungs [31]. Additionally, another finding in terms of the hemogram parameters in this study that supports this effect of vitamin D in viral infections was the higher lymphocyte count at a normal range in the AS group when compared with the IS group, since lymphocytes are required to support the host in the immune defense against virus infections [32].

Regarding weight loss and a reduction in BMI, we observed intense weight loss in both groups (As and IS) without significant differences between them, and in line with our findings, Rodrigues and coworkers found a positive correlation between concentrations of vitamin D and weight loss after 6 and 12 months of bariatric surgery [33]. This fact may be justified by the release of the possible stock of vitamin D that is retained in the adipose tissue, which increases the serum concentrations of this vitamin and its bioavailability [33,34].

Therefore, vitamin D adequacy has a significant effect on weight control, and a growing body of evidence suggests that vitamin D is involved in numerous processes in human adipose tissue, and this evidence mainly comes from studies of human adipocytes and adipose tissue, which is related to the effects of vitamin D on adipogenesis, energy homeostasis, and inflammation. Additionally, these effects of vitamin D are mediated through the VDR [35]. After stimulation with 1,25(OH)_2_D, VDR expression is increased in the adipose tissues of individuals with obesity, and it is also higher in the visceral preadipocytes of these individuals during the differentiation process. High adiposity is also associated with increased VDR messenger RNA (mRNA) levels and decreased CYP27B1 mRNA in adipose tissues (visceral and subcutaneous) [36].

However, despite the fact that an RYGB reduces caloric intake in the immediate postoperative period due to the reduced gastric capacity, decreased hunger, and increased satiety, for some patients, the caloric intake gradually increases and hence contributes to weight regain to levels similar to presurgery obesity in terms of body composition, the return of metabolic disorders associated with body fat accumulation [37], and a high percentage of vitamin D inadequacy. The inadequacy of this vitamin, in part, can be explained by the presence of visceral adiposity in these individuals, considering the association between inadequate vitamin D nutritional status and the location where the fat is deposited [38].

The limitation of this study is that the sample size did not allow for a subgroup analyses on the 25(OH)D serum concentrations, whereby the concentrations would have been grouped into low level/suboptimal/sufficient subgroups. However, to date, no previous study has evaluated the association between vitamin D status with thyroid hormone profiles and immune inflammatory parameters in patients 6 months after an RYGB.

## 5. Conclusions

We found a significant reduction in TSH levels only in the group that achieved an adequate vitamin D nutritional status. Additionally, the adequacy group had a significantly lower BMI 12 months after surgery. According to these findings, the diagnosis of hypothyroidism in individuals with obesity and a vitamin D deficiency and the assessment of serum vitamin D concentrations in this population seem relevant. The analysis of one’s vitamin D nutritional status seems to be relevant as an adjuvant therapy in the weight loss process and as a protective factor for subclinical hypothyroidism.

We also observed a significant reduction in immune inflammatory markers such as the NLR and leukocyte count in the patients in the AS group 6 months after surgery, but no changes occurred in the patients in the IS group. In addition to that, the lymphocyte count was higher in the AS group, which suggested an additional improvement, specifically against viral respiratory tract infections.

More studies are needed to clarify the effect of reaching an adequate vitamin D nutritional status on weight loss after bariatric surgery in a longer follow-up period, such as 12 to 24 months after surgery, and on weight gain 5 years after surgery based on the hormonal findings observed in this study.

## Figures and Tables

**Table 1:** Baseline characteristics of both groups classified as adequate and inadequate according to nutritional status of vitamin D 6 months after surgery.

Characteristic	Inadequate (<30 ng/mL) *n* = 37	Adequate (≥30 ng/mL) *n* = 51	*p* Value
25(OH)D (ng/mL)	21.92 (7.66)	22.91 (8.90)	0.74
Vitamin D status			
Adequate (≥30 ng/mL)—no. (%)	8 (21.6%)	8 (15.7%)	0.43
Age—year	40.76 (9.22)	40.08 (9.90)	0.74
Male sex—no. (%)	17 (45.9%)	17 (33.3%)	0.23
Body mass index—kg/m^2^	43.84 (5.47)	44.31 (5.67)	0.53
Body weight (kg)	121.84 (19.45)	122.88 (25.26)	0.74
Hypothyroidism—no. (%)	5 (13.5%)	8 (15.7%)	0.77

25(OH)D—25-hydroxyvitamin D. Quantitative data are expressed as mean (SD), and qualitative data are expressed as *n* (% frequency).

**Table 2:** Changes in serum concentrations of 25(OH)D. Calcium metabolism and thyroid hormones at baseline and 6 months after surgery in both groups classified according to nutritional status of vitamin D 6 months after surgery.

Variables (Normal Range)	Inadequate (<30 ng/mL) *n* = 37			Adequate (≥30 ng/mL) *n* = 51			*p* (between)
	Baseline	6 Months	*p* (within)	Baseline	6 Months	*p* (within)	
25(OH)D (ng/mL) (30–100 ng/mL)	21.92 (7.66)	23.67 (4.27)	0.230	22.91 (8.89)	38.98 (13.93)	<0.001 *	<0.001 *
Calcium (mg/dL) (4.5–5.2 mg/dL)	4.37 (1.05)	4.52 (0.26)	0.530	4.52 (0.58)	4.67 (0.17)	0.090	0.008 *
PTH (pg/mL) (12–65 pg/mol)	43.82 (16.11)	43.76 (13.80)	0.990	46.36 (14.20)	41.09 (16.14)	0.040 *	0.352
TSH (µUI/mL) (0.45–4.50 µUI/mL)	3.09 (2.04)	2.84 (1.39)	0.540	3.01 (2.14)	2.22 (1.20)	0.017 *	0.020 *
FT_4_ (ng/dL) (0.7–1.48 ng/dL)	1.25 (0.26)	1.16 (0.21)	0.150	1.15 (0.19)	1.29 (0.90)	0.311	0.391
T_3_ (ng/dL) (70–210 ng/dL)	131.56 (38.19)	110.13 (22.88)	0.020 *	138.82 (39.16)	116.22 (26.42)	<0.001 *	0.212
Hypothyroidism—no. (%)	8 (24.3%)	5 (13.5%)		11 (21.6%)	8 (15.7%)		

25(OH)D—25-hydroxyvitamin D. PTH—parathyroid hormone. TSH—thyrotropin. FT_4_—free thyroxine. T_3_—total triiodothyronine. Mean (SD) (all variables). * Statistically significant.

**Table 3:** Inflammatory and immunological biomarkers change at 6 months after surgery in both groups classified according to nutritional status of vitamin D at 6-month follow up.

Variables (Normal Range)	Inadequate (<30 ng/mL) *n* = 37			Adequate (≥30 ng/mL) *n* = 51			*p* (between)
	Baseline	6 Months	*p* (within)	Baseline	6 Months	*p* (within)	
Uric acid (mg/dL) (2.6–7.2 mg/dL)	5.78 (1.46)	5.00 (1.20)	0.001 *	5.91 (1.28)	5.12 (1.42)	<0.001 *	0.69
Hs-CRP (mg/L) (<1 mg/L)	1.26 (3.07)	0.74 (0.76)	0.38	1.09 (0.93)	1.08 (4.05)	0.98	0.64
Leukocytes (mm^3^) (4000–10,000 mm^3^)	7053 (1666)	6529 (1526)	0.11	7488 (1496)	6427 (1691)	<0.001 *	0.68
Neutrophils (mm^3^) (1600–8000 mm^3^)	4235 (1580)	3406 (965)	0.009 *	4510 (1319)	3469 (1136)	<0.001 *	0.67
Lymphocytes (mm^3^) (1000–3900 mm^3^)	2081 (588)	1790 (532)	0.22	2408 (568)	2261 (695)	0.12	0.04 *
Platelet (10^3^/mm^3^) (150–450 10^3^/mm^3^)	257.32 (72.39)	241.20 (80.89)	0.26	271.24 (70.17)	253.4 (66.31)	0.051	0.46
NLR (1–3)	2.13 (0.82)	1.99 (0.62)	0.60	1.96 (0.73)	1.59 (0.47)	0.01 *	0.08

Hs-CRP—high-sensitivity C-Reactive Protein. NLR—neutrophil-to-lymphocyte ratio. Mean (SD) (all variables). * Statistically significant.

**Table 4:** Changes in weight loss (%) at 6 months and in body weight at 6 and 12 months after surgery in both groups classified according to nutritional status of vitamin D at 6-month follow up.

Variables	Inadequate (<30 ng/mL) *n* = 37				Adequate (≥30 ng/mL) *n* = 51			
	Baseline	6 Months	12 Months	*p*-Value	Baseline	6 Months	12 Months	*p*-Value
Weight (kg)	121.23 (19.36)	86.96 (16.03) ^a^	99.04 (18.23) ^a,b^	0.001	122.87 (25.30)	86.61 (20.81) ^a^	85.68 (20.82) ^a,b^	0.001
BMI (kg/m^2^)	43.56 (5.29)	31.17 (4.95) ^a^	35.04 (5.38) ^a,b^	0.001	44.34 (5.77)	31.31 (4.56) ^a^	31.51 (5.08) ^a,b^	0.001
Total weight loss (%)	-	28.51 (7.26)	29.34 (12.33)	0.078	-	29.44 (5.16)	31.21 (9.15)	0.107
Excess weight loss (%)	-	69.75 (20.27)	76.21 (31.62)	0.219	-	69.94 (15.62)	78.22 (28.6)	0.329

Mean (SD) (all variables). BMI—body mass index. ^a^ Significant difference compared to baseline time—intragroups analysis. ^b^ Significant difference between groups in the same time interval—intergroups analysis.

## Data Availability

The data used to support the findings of this study are available from the corresponding author upon request. Data is not publicly available due to privacy.
